# Factors Associated with Volunteer Activities and Sleep Efficiency in Older Adults with Hypertension: A Sequential Model Study

**DOI:** 10.3390/geriatrics6030089

**Published:** 2021-09-11

**Authors:** Ryoko Aonuma, Thomas Mayers, Katsuyoshi Mizukami, Kazutaka Aonuma, Hitomi Matsuda

**Affiliations:** 1 Graduate School of Comprehensive Human Sciences, Department of Human Care Science, University of Tsukuba, 1-1-1 Tennodai, Tsukuba 305-8575, Ibaraki, Japan; matsuda.hitomi.fn@u.tsukuba.ac.jp; 2 Department of Health Services Research, Faculty of Medicine, University of Tsukuba, 1-1-1 Tennodai, Tsukuba 305-8575, Ibaraki, Japan; mayers@md.tsukuba.ac.jp; 3 Medical English Communications Center, Faculty of Medicine, University of Tsukuba, 1-1-1 Tennodai, Tsukuba 305-8575, Ibaraki, Japan; 4 Graduate School of Comprehensive Human Sciences, Faculty of Health and Sport Sciences, University of Tsukuba, 1-1-1 Tennodai, Tsukuba 305-8575, Ibaraki, Japan; mizukami.katsuyos.ga@u.tsukuba.ac.jp; 5 Department of Cardiology, Faculty of Medicine, University of Tsukuba, 1-1-1 Tennodai, Tsukuba 305-8575, Ibaraki, Japan; kaonuma@md.tsukuba.ac.jp

**Keywords:** volunteering, sleep, older adults, hypertension, depression, sequential model

## Abstract

The purpose of this study was to examine, using a sequential model, factors associated with volunteer participation and sleep efficiency in Japanese older adults receiving treatment for hypertensive disease. A questionnaire survey was conducted to collect data on participant demographics, lifestyle, health status, and depression, and sleep activity monitors were used to objectively measure sleep status and sleep efficacy. Of the 167 respondents, the 59 being treated for hypertension were divided into two groups based on their participation in volunteering. Comparison between the groups showed significant differences in nocturnal awakening, sleep efficiency, and nap frequency. Volunteers had less nocturnal awakening, increased sleep efficiency, fewer naps, and decreased depression. Covariance structure analysis of the survey data and sleep measurements for hypertensive older adults in the volunteer group was performed by modeling the relationships between variables with a path diagram. Our model showed strong goodness of fit (χ^2^ test = 15.636, *p* = 0.111, GFI = 0.925, AGFI = 0.842, CFI = 0.925, RMSEA = 0.099). The findings of this study suggest that older adults with hypertension who participate in volunteer activities have less nocturnal awakening, improved sleep quality, and reduced risk of depression, and provides evidence to promote social participation in volunteering among older adults with hypertension.

## 1. Introduction

Hypertension, a lifestyle-related disease, is a major risk factor for cardiac and cerebrovascular disease. As the National Health and Nutrition Survey in Japan 2019 [1] revealed, a national prevalence as high as 61.3% in Japanese people aged 65–74 and 65.6.3% aged 75 and over, Japanese national health promotion measures often target hypertension as a means of extending healthy life expectancy [2].

A large number of studies highlight the strong association between hypertension and sleep disorders [3]. A large-scale population-based cohort study from Taiwan showed that hypertension was significantly higher among those with sleep disorders than controls [4]. In Japan, one in three older adults complain of insomnia [5], which is a sleep disorder linked to both hypertension [6] and depression [7]. Sleep has been reported as an important mediating factor between stress and hypertension [8], and stress and mental health [9]. Thus, interventions to improve quality of sleep for older adults may have health benefits by ameliorating hypertension and decreasing depressive/mental health symptoms.

Volunteering was shown to have numerous diverse health benefits for adults [10] and, specifically in older adults, social interactions, such as those experienced with volunteering, improve the sense of self-worth and willingness to live while suppressing depression [11]. Furthermore, it was reported that participation in volunteer activities reduces the risk of hypertension [12,13].

Taken together, this evidence suggests that volunteering may have positive physical and psychological effects for older adults and that sleep may be an important mediating factor. The aim of this study, therefore, was to investigate the association between volunteering and sleep in older adults with hypertension. Our findings indicate that older adults with hypertension who participate in volunteer activities have high sleep efficiency and lower levels of depression.

## 2. Materials and Methods

### 2.1. Participants

This cross-sectional study involved Japanese older adults (average age 77.27 ± 5.54) with hypertension who were affiliated with the Senior Citizens’ Clubs in Ibaraki and Okinawa prefectures. Verbal and written explanations of the research were given to the directors of the six senior citizens’ clubs, who, in turn, helped to recruit willing participants for this study in advance. Study participants included those older adults who understood the content of the survey and the study protocol, including sleep measurement using ActiSleep monitors (ActiGraph, Pensacola, FL, USA), and who gave their informed consent for participation.

### 2.2. Survey

A questionnaire survey was conducted to collect participant demographic data, daily life information, lifestyle, sleep status, and health status. The survey was conducted annually over a 4-year period (2014–2017) between the months of February and May to minimize any regional differences in weather.

The demographic data collected were sex, age, and living arrangements. Daily life data collected were frequency of going out and daily activities, including housework, volunteering, hobbies, lessons, and exercise classes. Regarding lifestyle, participants were asked about smoking and drinking habits. Items on sleep status, frequency of naps, and nocturnal awakening for urination were also included. Health status measurements comprised body mass index (BMI), depression status (Geriatric Depression Scale [GDS]-15 short form) [14], and health-related quality of life (HR-QOL), which was measured using the Short Form 8 (SF-8) [15]. The GDS-15, designed specifically to assess depressive symptoms in older adults, renders scores between 0–15, with a score over 5 indicating the possibility of depression. The SF-8 includes subordinate items measuring various aspects of physical and mental health. By weighting each item, summary scores representing physical health (PCS) and mental health (MCS) are rendered. Scoring is based on national standard values. In addition, the presence or absence of sleep disorders and diabetes mellitus (DM) were investigated.

### 2.3. ActiSleep Measurement

The ActiSleep-BT activity monitor (ActiGraph, Pensacola, FL, USA) was used to objectively measure the participants’ nighttime sleep status. The ActiSleep-BT is a lightweight device, worn on the wrist, that determines awakening/sleep by measuring the amount of activity. Body movement during sleep is calculated as an activity count and the resulting data is highly compatible with sleep polysomnography to an accuracy of 85% [16]. The ActiSleep analysis software, ActiLife version 6 (ActiGraph, Pensacola, FL, USA), was used for data analysis. Total sleep time, sleep latency, awakening time, and sleep efficiency were analyzed.

Additionally, to understand the participants’ life rhythms during the measurement period, they were asked to complete a “life rhythm table” in which they entered the corresponding times and details for items, such as bedtime, wake-up time, meals, conversations, nocturnal urination, and naps. 

### 2.4. Data Analysis

A simple tabulation was performed for the questionnaire items relating to participant demographics, lifestyle, health status, sleep status, etc. Participants were divided into two groups, Volunteer and Non-Volunteer, based on their participation in volunteer activities. Fisher exact test, χ^2^ test, and Mann–Whitney U test were performed to compare the two groups. The significance level was set to 5% (*p* < 0.05).

Covariance structure analysis was performed with the results of the questionnaire survey and the ActiSleep measurements by modeling the relationships between variables with a path diagram. A covariance structure model integrates the relationship between constructs based on a hypothesis and, by repetition and reanalysis, the original model is iterated towards a model that fits the data [17]. The goodness-of-fit indices (GFI), adjusted GFI (AGFI), normed fit index (NFI), comparative fit index (CFI), root mean square error of approximation (RMSEA), and Akaike’s information criterion (AIC) were used to compare how well the model fits the observed results [18].

Direct effect and overall/total effect were examined to establish the effect of the causative variables on the result variable and evaluate the total direct and indirect effects [19]. IBM SSPS Amos version 21 (IBM, Armonk, NY, USA) was used for the path analysis and the significance level was set to 5% (*p* < 0.05).

### 2.5. Ethical Statement

This study was conducted with the approval of the ethics committee at the University of Tsukuba Hospital (Approval No. 932-4). Thorough explanations of the purpose and methods of the study were given to the regional directors of the Senior Citizens’ Club and all participants and written informed consent for participation was obtained. 

## 3. Results

### 3.1. Participant Demographics

None of the 167 recruited participants withdrew their consent for participation during the survey or measurements (participation rate 100%). Of those 167 participants, 59 self-reported that they were being treated for hypertension. Data regarding basic demographics, daily activities, lifestyle, sleep status, and health status of the 59 participants with hypertension are shown in Table 1 and Table 2.

**Figure 1:**
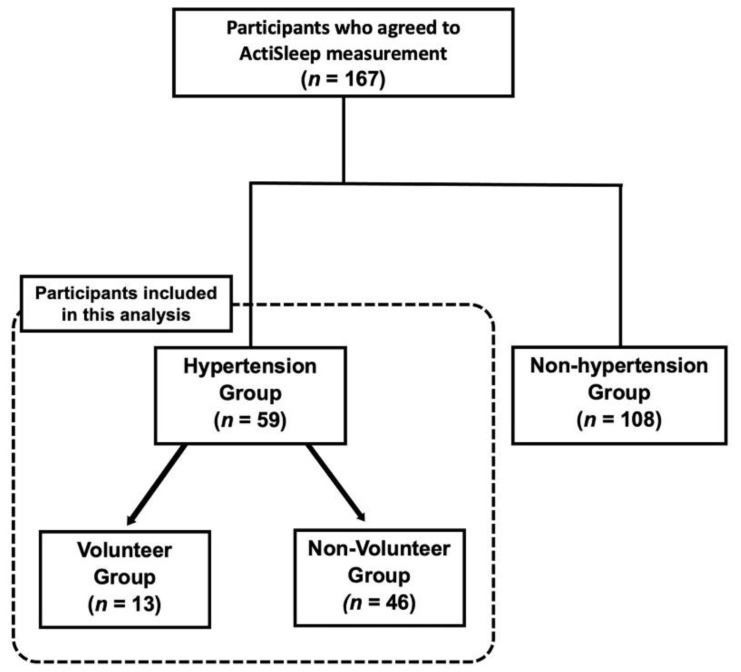
Flowchart of participation in the study.

Of those 59 participants, the 13 (22.0 %) who were currently actively engaged in volunteering were assigned to the Volunteer Group and the remaining 46 (78.0%) were assigned to the Non-Volunteer Group. A flowchart of participation is shown in Figure 1. The average age was 77.27 (SD ± 5.54 years), 34 (57%) were female, 43 (74.1%) had a spouse, and 53 (93.0%) had children. Nine (15.8%) were living alone, while forty-eight (84.2%) lived with others (spouse or family). There were no significant differences regarding basic demographics between the two groups although the average age of volunteers (75.85 ± 4.26) was slightly lower than non-volunteers (77.67 ± 5.83).

### 3.2. Daily Activities

Regarding the activities of daily living, 34 people (57.6%) did housework, 28 (47.5%) engaged in hobbies/lessons, and 16 (27.1%) did exercise classes. Compared to volunteers, non-volunteers were more likely to do housework (*p* = 0.128) but less likely to engage in hobbies/lessons (*p* = 0.348), and exercise classes (*p* = 0.154). The average number of days per week that the participants went out (frequency of going out) was 5.68 (SD ± 1.69) and was slightly higher among volunteers (6.23 ± 1.30) than non-volunteers (5.52 ± 1.76; *p* = 0.203). Only two people (3.4%) were smokers, and six people (10.3%) drank alcohol.

### 3.3. Sleep Status

In regard to sleep status, the average total sleep time (minutes) was 364.95 (SD ± 99.07; range: 101–649), sleep onset latency (minutes) was 6.47 (SD ± 5.37; range: 1–38), awakening time (minutes) was 89.2 (SD ± 46.17; range: 8–241.33), and sleep efficiency (%) was 79.39% (SD ± 8.99; range: 56.96–94.75). Volunteers had a longer total sleep time (389.54 ± 130.81) than non-volunteers (358.00 ± 88.68), although the difference was not significant (*p* = 0.156). There was, however, a significant difference (*p* = 0.003) in the awakening time between the two groups, with that of the volunteers being much shorter (59.92 ± 46.63) than non-volunteers (97.47 ± 43.01). There was also a significant difference (*p* = 0.001) in sleep efficiency between volunteers (86.32 ± 6.79) and non-volunteers (77.43 ± 8.60). The nap frequency (days/week) was 3.08 (SD ± 2.60) and there was a significant (*p* = 0.009) difference between volunteers (1.46 ± 2.18) and non-volunteers (3.54 ± 2.54). The number of people who woke up at night to urinate was 49 (83.1%), with no significant difference between the two groups.

### 3.4. Health Status

With respect to health status, the average BMI was 24.38 (SD ± 3.11; range: 17.63–32.37) while 24 out of 59 participants had some level of obesity (BMI ≥ 25). The mean GDS score was 2.29 (SD ± 2.69; range: 0–11), indicating a very low prevalence of depressive symptoms. The average PCS summary score was 47.49 (SD ± 7.38; range: 25.43–56.30), which was higher than the reference value (44.78 ± 9.18). While not significant (*p* = 0.351), PCS scores were slightly higher in volunteers (50.04 ± 3.49) than non-volunteers (46.76 ± 8.03). The mean MCS summary score was 50.13 (SD ± 5.96; range: 29.93–62.96), which was comparable with the reference value (50.95 ± 6.95). Again, there was no significant difference (*p* = 0.314) in the MCS summary score between the two groups, the Volunteer Group’s average score (51.77 ± 3.53) slightly exceeded the Non-Volunteer Group (49.66 ± 6.44) and the reference value. Among the 59 participants, there were five people with sleep disorders and five with DM.

### 3.5. Model to Describe the Relationship between Factors Related to Volunteer Participation, Hypertension, and Sleep in Older Adults

With reference to the items that showed some significant difference between volunteers and non-volunteers (awakening time, total sleep time, sleep efficiency, nap frequency, and GDS, PCS, and MCS scores), we created and tested a covariance structure analysis model to describe the relationship between factors related to volunteer participation with high hypertension and sleep in older adults.

**Figure 2:**
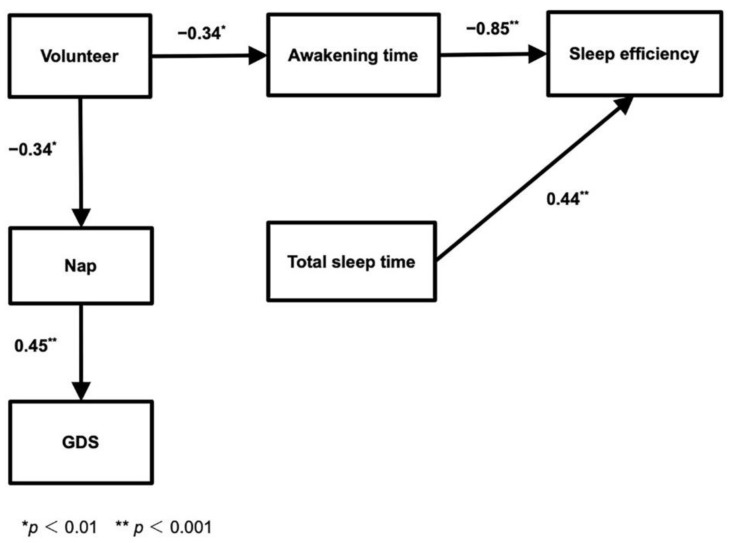
Model: Covariance structure model examining the relationship between quality of sleep and volunteer participation in older adults with hypertension. Non-significant paths were removed. Means and SDs of nap, GDS, awakening time, sleep efficiency, and total sleep are not shown here. Goodness of fit indices: χ^2^ = 15.636; GFI = 0.925; NFI = 0.919; RMSEA = 0.099; *p* = 0.111; AGFI = 0.842; CFI = 0.968; AIC = 37.636.

The model, shown in Figure 2, is a sequential model in which each variable was significant (*p* < 0.01). Table 3 shows the data that forms the basis of our model. Regarding goodness of fit, the model showed high statistical significance (χ^2^ = 15.636; *p* = 0.111) and the various indices indicated that the model has high compatibility (GFI = 0.925; AGFI = 0.842; NFI = 0.919; CFI = 0.968; RMSEA = 0.099; AIC = 37.636).

The model revealed a direct effect for volunteers since the path coefficient from “volunteer” to “awakening time” was −0.34, and the path coefficient from “awakening time” to “sleep efficiency” was −0.85, meaning that a shorter awakening time was associated with higher sleep efficiency. Volunteers had fewer naps than non-volunteers and, in the model, the path coefficient from “volunteer” to “nap” was −0.34 and 0.45 from “nap” to “GDS”, suggesting that higher frequency of napping was associated with higher GDS scores. The path coefficient from “total sleep time” to “sleep efficiency” was 0.44, suggesting that a longer total sleep time is associated with higher sleep efficiency.

As for overall effect, the path coefficient from “volunteer” to “sleep efficiency” via “awakening time” was 0.29 (volunteers did show higher sleep efficiency) while the path coefficient from “volunteers” to “GDS” via “nap” was −0.15, indicating a possible explanation for the lower GDS scores in volunteers. Thus, our model (Figure 2) was adopted for its high compatibility and explanatory power. The model intimates that older adults with hypertension who participate in volunteer activities have higher sleep efficiency and lower levels of depression.

## 4. Discussion

### 4.1. Sleep

Previous studies have reported that older adults with hypertension have significantly lower sleep efficiency [20]. The average sleep efficiency of those aged from 60 to 80 years was reported as 79.2–81.4% [21], while the average value of sleep efficiency of the volunteers in our study was 86.32%. Therefore, even though the volunteers in our study had hypertension, they exhibited higher-than-average sleep efficiency.

Regarding nocturnal awakening, it is reported that physical activity increases deep sleep, which reduces nocturnal awakening and improves insomnia [22]. The volunteers’ SF-8 PCS summary scores were, on average, higher than non-volunteers, indicating that volunteers had higher physical functioning. The average PCS summary score of the Volunteer Group in our study was higher than the Japanese national reference value for the same age group (50.04 ± 3.49 and 44 ± 9.18, respectively). Furthermore, the volunteers’ PCS summary score was higher than the reference value for individuals with one chronic disease (49.24 ± 6.74). Thus, because the PCS summary score was relatively high among volunteers, we speculate that physical activity was enhanced and nocturnal awakening time curtailed through active participation in volunteer activities.

As for the relationship between volunteering and physical activity, Mitsuishi et al. reported that the amount of physical activity increased with volunteering [23] and suggested that it is necessary to investigate the type and frequency of volunteer activity in order to better understand this association. In addition, older adults with hypertension are characterized as having a “nondipper” type of nocturnal blood pressure (BP) in which nocturnal BP fluctuations inhibit the usual decrease in BP during the nighttime [24]. In a previous report, which used ActiGraph to measure the amount of physical activity during sleep, Kario et al. suggested that for those with the nondipper BP type, frequent awakening occurs due to an increased amount of nocturnal physical activity, which affects the quality of sleep [25]. Pasqualini et al. further reported that older adults with nondipper BP type have significantly lower sleep efficiency and more awakenings than those with the dipper type [26]. From these findings, it is clear that older adults with hypertension are more susceptible to nocturnal awakenings and poor sleep quality. However, the results of our previous study showed that older adults with hypertension who participate in volunteer activities have less frequent nocturnal awakenings [27]. Likewise, in the current study, the results of the overall effect of the model showed that even with hypertension, volunteering promoted high sleep efficiency through reduced nocturnal awakening. Therefore, the model of this study adds further evidence to the benefits of volunteering on sleep efficiency and control of hypertension in older adults.

### 4.2. Depression

In the current study, volunteers took naps significantly less frequently than non-volunteers. Comparing the daily activities of the two groups, non-volunteers had a higher rate of participation in housework but less engagement in lessons and exercise classes. On the other hand, volunteers had a higher average PCS summary score and frequency of outings and thus they can be described as active older adults. While the survey did not ask the type and frequency of volunteer activities that the participants were engaged in, we hypothesize that the reduced nap time was due to volunteering.

Covariance structure analysis showed a direct effect of nap frequency on GDS scores, with a higher nap frequency being associated with higher GDS scores. In this study, nap duration was not measured; however, according to previous reports, men who take long naps (54 min or more) have higher levels of depression [28] and, likewise, women who are depressed frequently take naps [29] since excessive daytime sleepiness is associated with depression [30]. However, while there seems to be a relationship between napping and depression, the beneficial effects of napping for older adults have also been reported [31]. The findings of these previous studies alongside our results suggest that, for older adults with hypertension, it is necessary to consider appropriate nap routines to facilitate continued volunteering while guarding against depressive symptoms. The overall effect of the model in this study showed that volunteer participation was associated with a reduction in GDS scores. Therefore, volunteering could be said to have a protective effect against depression.

### 4.3. Volunteer Participation in Older Adults

The results of a 2017 survey by the Japan NGO Council on Ageing, indicate that older adults want to stay healthy and are interested in volunteer activities and societal contribution [32]. According to Fujiwara et al. [33], most studies that have analyzed the direct effects of volunteering on health have used psychological measures, such as depression, as the objective variable but few studies have examined the effects on physical health [34]. As for physical effects, volunteering was shown to reduce the decline in the activities of daily living [35] and reduce the risk of hypertension [12]. In addition, volunteering was reported to improve life expectancy [36]. The results of the above-mentioned studies, however, are mainly based on questionnaire surveys and not from objective data. In contrast, our study included objective instrumental measurement and analysis of participant sleep status. In addition, there are no reports that have clarified the associations between sleep and GDS scores in older adult volunteers with hypertension.

The model of this study, therefore, is useful for promoting participation in volunteer activities which are said to impart self-esteem and, even in the later years of life, the feeling that they can still be of use to others as well as alleviating the loneliness associated with old age [37].

### 4.4. Limitations and Future Directions

Some limitations of this study must be acknowledged. Firstly, the small number of participants, particularly in the volunteer group, limits the generalizability of the findings. Secondly, participants were identified as hypertensive solely on the basis of their self-reporting; they were asked whether or not they were currently under treatment for hypertension, but no detailed data were gathered on the type of hypertension, disease duration and severity, nor the kind of antihypertensive drugs being used for treatment, therapeutic adherence, and so on. Therefore, future studies that include larger numbers of participants, objective BP measurements, and more detailed investigation of the patients’ hypertensive disease would perhaps strengthen and add granularity to these findings. As napping was reported to be associated with both positive and negative effects, future studies are necessary to evaluate what constitutes appropriate/optimal napping. Additionally, future research is needed to develop a model that incorporates the effects of other daily activities such as hobbies and exercise activities.

## 5. Conclusions

The findings of this study suggest that older adults with hypertension who participate in volunteer activities have less nocturnal awakening and improved sleep quality. Furthermore, our finding that volunteers had lower GDS scores indicates that volunteering might reduce the risk of depression. Thus, although hypertension is a disease with a high incidence among older adults in Japan, we found that volunteer activities provided sleep and mental peace of mind that were perhaps useful in controlling blood pressure. The results of this study add to a growing body of evidence that volunteering has diverse beneficial effects for both the physical and mental health of older adults and encouraging participation in volunteer activities could help to extend their healthy life expectancies and improve their overall wellbeing.

## Figures and Tables

**Table 1:** Comparison of basic demographics and lifestyle habits of volunteers and non-volunteers.

				*N* = 59
	Entire Cohort	Volunteers	Non-Volunteers	*p*
	*N* = 59	*N* = 13 (22.0)	*N* = 46 (78.0)	
Sex				0.762 ^†^
Male	25 (42.4)	6 (46.2)	19 (41.3)	
Female	34 (57.6)	7 (53.8)	27 (58.7)	
Age	77.27 ± 5.54	75.85 ± 4.26	77.67 ± 5.83	0.322 ^‡^
Spouse				0.480 ^†^
Yes	43 (74.1)	11 (84.6)	32 (71.1)	
No	15 (25.9)	2 (15.4)	13 (28.9)	
Living arrangements				0.668 ^†^
Living alone	9 (15.8)	1 (7.7)	8 (18.2)	
Not living alone	48 (84.2)	12 (92.3)	36 (75.0)	
Children				1.000 ^†^
Yes	53 (93.0)	12 (92.3)	41 (93.2)	
No	4 (7.0)	1 (7.7)	3 (6.8)	
Housework				0.128 ^†^
Yes	34 (57.6)	5 (38.5)	29 (63.0)	
No	25 (42.4)	8 (61.5)	17 (37.0)	
Hobbies/lessons				0.348 ^†^
Yes	28 (47.5)	8 (61.5)	20 (43.5)	
No	31 (52.2)	5 (38.5)	26 (56.5)	
Exercise class				0.154 ^†^
Yes	16 (27.1)	6 (46.2)	10 (21.7)	
No	43 (72.9)	7 (53.8)	36 (78.3)	
Frequency of going out	5.68 ± 1.69	6.23 ± 1.30	5.52 ± 1.76	0.203 ^‡^
Smoking habits				1.000 ^†^
Yes	2 (3.4)	0	2(4.3)	
No	57 (96.6)	13 (100)	44 (95.7)	
Drinking habit				0.119 ^†^
Yes	6 (10.3)	3 (23.1)	3 (6.7)	
No	52 (89.7)	10 (76.9)	42 (93.3)	

^†^ χ^2^ test, ^‡^ Mann–Whitney U.

**Table 2:** Comparison of sleep status and health status between volunteers and non-volunteers.

				*N* = 59
	Entire Cohort	Volunteers	Non-Volunteers	*p*
	*N* = 59	*N* = 13 (22.0)	*N* = 46 (78.0)	
Total sleep time (min)	364.95 ± 99.07	389.54 ± 130.81	358.00 ± 88.68	0.156 ^‡^
Sleep latency (min)	6.47 ± 5.37	7.85 ± 9.47	6.09 ± 3.56	0.578 ^‡^
Awakening time (min)	89.2 ± 46.17	59.92 ± 46.63	97.47 ± 43.01	0.003 *^‡^
Sleep efficiency (%)	79.39 ± 8.99	86.32 ± 6.79	77.43 ± 8.60	0.001 *^‡^
Nap frequency	3.08 ± 2.60	1.46 ± 2.18	3.54 ± 2.54	0.009 *^‡^
Nocturnal urination				1.000 ^†^
Yes	49 (83.1)	11 (84.6)	38 (82.6)	
No	10 (16.9)	2 (15.4)	8 (17.4)	
Sleeping disorder				1.000 ^†^
Yes	5 (8.5)	1 (7.7)	4 (8.7)	
No	54 (91.5)	12 (92.3)	42 (91.3)	
BMI	24.38 ± 3.11	23.83 ± 2.89	24.53 ± 3.18	0.564 ^‡^
GDS	2.29 ± 2.69	1.62 ± 1.33	2.48 ± 2.95	0.736 ^‡^
PCS	47.49 ± 7.38	50.04 ± 3.49	46.76 ± 8.03	0.351 ^‡^
MCS	50.13 ± 5.96	51.77 ± 3.53	49.66 ± 6.44	0.314 ^‡^
Diabetes				0.576 ^†^
Yes	5 (8.5)	0	5 (10.9)	
No	54 (91.5)	13(100)	41 (88.4)	

^†^ χ^2^ test, ^‡^ Mann–Whitney U test; * Statistically significant value.

**Table 3:** Data describing the relationship between quality of sleep and volunteer participation in older adults with hypertension that form the basis of the sequential model shown in Figure 2.

Factors						
			Estimated Value	Standard Error	Statistical Test	*p*
Awakening Time	<−	Volunteering	−37.551	13.638	−2.753	0.006
Naps	<−	Volunteering	−2.082	0.768	−2.711	0.007
Sleep efficiency	<−	Awakening Time	−0.166	0.013	−23.541	**
GDS	<−	Naps	0.465	0.122	3.819	**
Sleep efficiency	<−	Total sleep time	0.04	0.003	12.231	**
**Path Coefficient (Direct Effect**)						
			**Estimated Value**			
Awakening Time	<−	Volunteering	−0.34			
Naps	<−	Volunteering	−0.34			
Sleep efficiency	<−	Awakening Time	−0.85			
GDS	<−	Naps	0.45			
Sleep efficiency	<−	Total sleep time	0.44			
**Standardized Total Effect**						
			**Volunteering**			
Sleep efficiency			0.29			
GDS			−0.15			

** *p* *<* 0.001.

## Data Availability

The data presented in this study are available in Table 1, Table 2 and Table 3.
